# Multiple Gene Transfer and All-In-One Conditional Knockout Systems in Mouse Embryonic Stem Cells for Analysis of Gene Function

**DOI:** 10.3389/fcell.2022.870629

**Published:** 2022-03-28

**Authors:** Teruhiko Suzuki, Satoko Takagi, Takahiko Hara

**Affiliations:** ^1^ Stem Cell Project, Tokyo Metropolitan Institute of Medical Science, Tokyo, Japan; ^2^ Graduate School of Medical and Dental Sciences, Tokyo Medical and Dental University, Tokyo, Japan; ^3^ Graduate School of Science, Department of Biological Science, Tokyo Metropolitan University, Tokyo, Japan

**Keywords:** embryonic stem cells, gene targeting, conditional knockout, homologous recombination, gene function

## Abstract

Mouse embryonic stem cells (ESCs) are powerful tools for functional analysis of stem cell-related genes; however, complex gene manipulations, such as locus-targeted introduction of multiple genes and conditional gene knockout conditional knockout, are technically difficult. Here, we review recent advances in technologies aimed at generating cKO clones in ESCs, including two new methods developed in our laboratory: the simultaneous or sequential integration of multiple genes system for introducing an unlimited number of gene cassettes into a specific chromosomal locus using reciprocal recombinases; and the all-in-one cKO system, which enables introduction of an EGFP reporter expression cassette and FLAG-tagged gene of interest under an endogenous promoter. In addition, methods developed in other laboratories, including conventional approaches to establishment of cKO cell clones, inducible Cas9-mediated cKO generation, and cKO assisted by reporter construct, invertible gene-trap cassette, and conditional protein degradation. Finally, we discuss the advantages of each approach, as well as the remaining issues and challenges.

## Introduction

Gene knockout (KO) technology has made a substantial contribution to knowledge of gene function. KO mice and KO cells prepared from them are frequently used for analysis of gene function in mammalian cells; however, generation of KO mice requires extended periods of time and cumbersome processes, including isolation of gene-targeted mouse embryonic stem cell (ESC) clones, production of chimera mice carrying the KO ESCs, establishment of germline-transmitted heterozygous mice, and cross-breeding of the heterozygous mice. Preparation of genetically disrupted cells is an alternative approach for analysis of gene function *in vivo*; however, it is also challenging, due to the technical difficulties involved in gene targeting. Recent developments in genome editing technologies have addressed these issues and greatly accelerated the molecular analysis of gene function (Gaj et al., 2013; Gupta and Musunuru 2014). CRISPR/Cas9 is the most popular genome editing system because of its high efficiency and easy design/implementation. CRISPR/Cas9 generates a double strand break (DSB) at the target site, which is repaired by the error-prone non-homologous end joining (NHEJ) process, resulting in the introduction of insertion/deletion mutations and consequent target gene disruption. CRISPR/Cas9-induced gene disruption is relatively efficient in mammalian cells and has greatly reduced the time and cost required for molecular analysis of gene function. Nevertheless, simple mutagenesis by genome editing technology is not suitable for analysis of lethal genes, which are essential for cell growth, survival, or maintenance of the undifferentiated status of stem cells. Gene knockdown with short interference RNA (siRNA or shRNA) is often applied in these cases; however, these knockdown systems often do not completely suppress target gene expression, which can lead to inconclusive results.

The conditional knockout (cKO) approach, first reported by Gu et al. (1994), is a useful way to study genes difficult to investigate using other approaches. cKO cells are usually generated using recombinases, such as Cre and FLP, in combination with their respective recognition sequences, loxP and FRT. The coding exon(s) of the target gene is/are flanked by these recognition sequences, and their corresponding recombinases are conditionally expressed to induce gene KO in specific cells. Definitive experimental results are expected, as the genetic disruption induces complete elimination of target gene expression. While cKO cells can represent an ideal option, their construction requires targeting of all alleles in each cell. Therefore, few cell lines are suitable for establishing cKO clones, since most are hyperploid and exhibit low homologous recombination efficiency. To overcome these challenges, various cKO methods have been developed with the aid of genome editing technology. In this review, we provide an overview of recent advances in the development of cKO strategies, particularly the all-in-one cKO system, as well as their advantages and issues that need to be addressed.

## cKO Cell Establishment Using a Conventional Strategy

**FIGURE 1:**
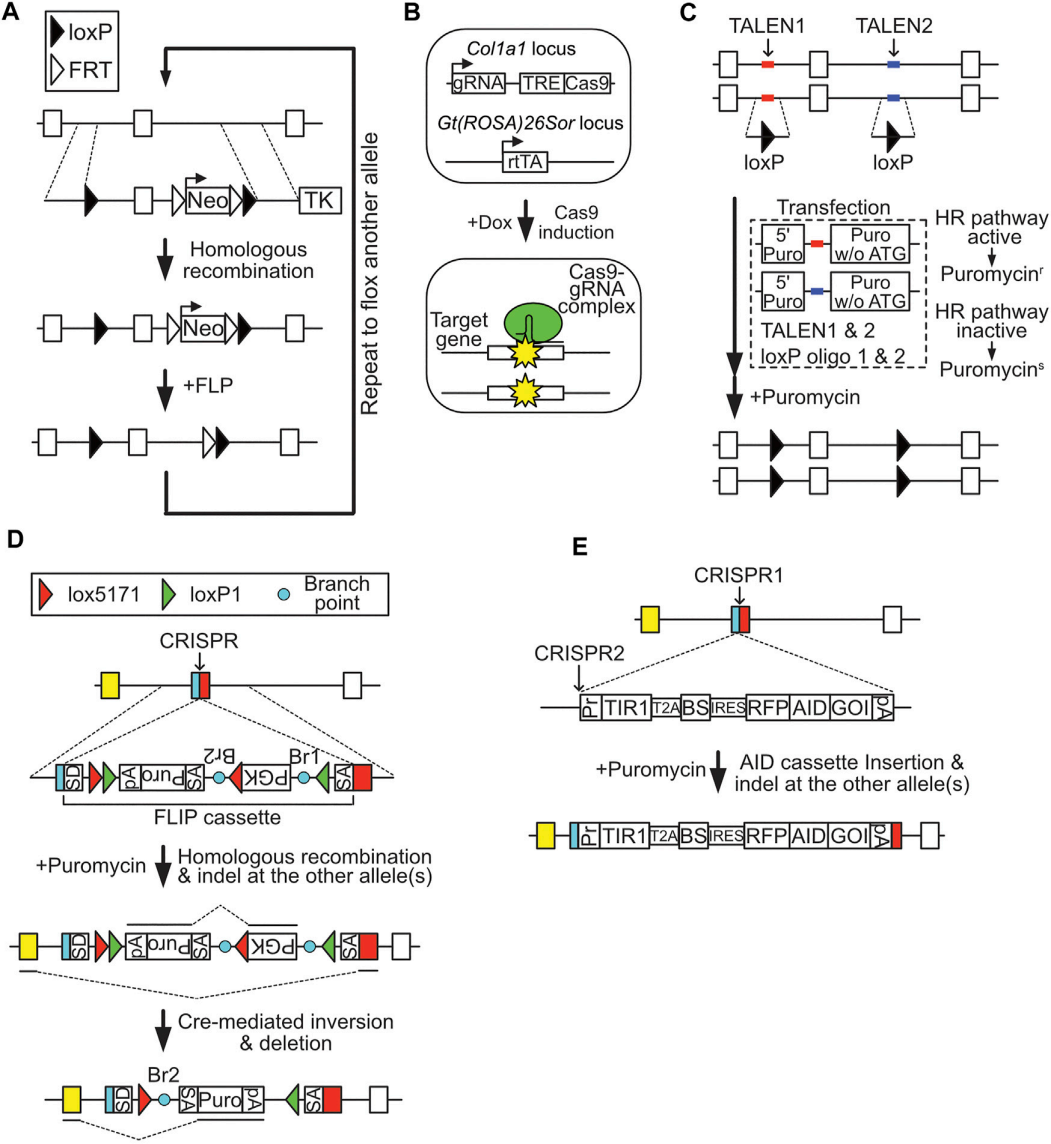
Methods for producing conditional knockout (cKO) cells. **(A)** Conventional cKO method. Filled triangle, loxP site; empty triangle, FRT site. **(B)** Inducible Cas9-mediated cKO method. **(C)** Reporter construct-assisted cKO method. **(D)** cKO using a FLIP cassette. SD, splicing donor sequence; SA, splicing acceptor sequence; Br, branch point; green triangle, loxP1 site; red triangle, lox5171 site; pA, polyadenylation signal. **(E)** cKO using the AID system.

Mouse ESCs are useful for preparing cKO cells, since ESCs have a normal karyotype and relatively high homologous recombination rates compared with conventional cell lines. The functions of various genes involved in stemness, cell growth, and survival have been clarified using cKO ESCs (Dejosez et al., 2008; Dovey et al., 2010; Lu et al., 2014). The conventional procedure for preparation of cKO ESCs involves introduction of a targeting vector containing positive-selection marker genes (e.g., a neomycin resistance gene) flanked by FRT sites, a negative-selection marker (e.g., herpes simplex virus-derived thymidine kinase), and a coding exon (or exons) of the target gene, flanked by loxP sites, into ESCs (Figure 1A). Targeted ESC clones can be isolated by positive-negative selection with 5–10% efficiency (Johnson et al., 1989). After isolation of a targeted clone, the positive-selection marker gene is removed by transient expression of FLP to retain normal expression of the target gene. These targeting processes are then repeated for the other allele. Therefore, a total of four cloning steps are required to establish cKO ESC clones. Due to this time-consuming process, establishment of cKO cells has not been a popular choice for analysis of gene function, despite its numerous advantages. Moreover, prolonged culture of ESCs for repeated cloning may compromise their undifferentiated characteristics. To avoid this concern, one could breed ESC-derived heterozygous mice, although this procedure requires much longer time.

## Inducible Cas9-Mediated cKO

Dow et al. (2015) reported a cKO system based on CRISPR/Cas9-induced gene disruption (Figure 1B). To enable conditional knockout of the target gene, a guide RNA expression cassette and doxycycline (Dox)-inducible Cas9 cassette were inserted to the safe harbor locus (genetically reliable locus), *Col1a1*, of ESCs stably expressing reverse tetracycline transactivator (rtTA). Using this system, approximately 70% of cells displayed biallelic frame-shift mutation of the target gene in a Dox-dependent manner. Moreover, this technique can induce simultaneous conditional knockout of two target genes with 40–50% efficiency. CRISPR/Cas9-mediated cKO is also applicable in mice. This system greatly reduces the time and labor required to generate cKO ESCs, as well as mice, since it allows one-step preparation of cKO cells. Nevertheless, cells with in-frame mutations, which may behave as normal cells, cannot be eliminated due to the principles underlying this system, which relies on NHEJ-dependent mutagenesis.

## Reporter Construct-Assisted cKO

DNA DSBs at the targeting site greatly enhance rates of homologous recombination (Donoho et al., 1998). Based on this mechanism, several genome editing technology-assisted methods have been developed for efficient cKO cell cloning. Flemr and Bühler introduced two homozygous loxP sites simultaneously *via* transfection of single-strand oligo-DNA, composed of a loxP sequence and 40 bp homology arms, TAL effector nucleases (TALENs) designed to target the site of interest, and a recombination reporter construct, which contained a 5′ puromycin-resistance gene fragment and a TALENs target sequence, followed by a full-length puromycin-resistance gene without a start codon (Figure 1C) (Flemr and Bühler 2015). If homologous recombination mechanisms are active in the transfected cells, TALENs-induced DSB of the reporter construct results in generation of an intact puromycin-resistance gene *via* homologous recombination. Using this method, these researchers successfully generated cKO ESCs by targeting two loxP oligo-DNA molecules on both alleles in a single step; however, this approach still requires extensive screening to obtain correct clones, due to the low efficiency (approximately 4%) of targeting of all four sites. Use of CRISPR/Cas9 instead of TALENs may improve the efficiency.

## cKO *via* Invertible Gene-Trap Cassette

Andersson-Rolf et al. used the Cre-regulated invertible gene-trap cassette (FLIP cassette), which relates to the gene trap tool originally developed by Melchner’s laboratory for preparing cKO cells (Schnütgen et al., 2005). The FLIP cassette contains a puromycin expression unit, flanked by loxP1 and lox5171 sites, in the middle of an artificial intron sequence (Figure 1D) (Andersson-Rolf et al., 2017). To produce cKO cells, the FLIP cassette is inserted into a coding exon of a target gene *via* homologous recombination, with the assistance of CRISPR/Cas9 designed for the target site. The resulting targeted clones can be cKO cells, as CRISPR/Cas9 both assists in targeting the FLIP cassette and destroys untargeted allele(s) of the target gene *via* NHEJ-dependent mutagenesis. Transient expression of Cre in the targeted clone induces inversion of the FLIP cassette and knocks out the target gene by switching the splicing donor/acceptor structure of the artificial intron. The authors produced *Ctnnb1* cKO ESC clones using this method, and verified the loss of ESC dome-like morphology on introduction of Cre. The CRISPR/Cas9 vector designed in the coding exon introduces the FLIP cassette in one allele, while it could also disrupt the other allele of the gene. This smart system is applicable in both ESCs and aneuploid cells, as homologous recombination of the FLIP cassette in one allele is sufficient to generate cKO cells. Nevertheless, the system still requires relatively extensive screening, since the efficiency is around 6% (4 FLIP/- clones out of 64 isolated clones in the case of ESC screening for *Ctnnb1* cKO cells). Further, gene expression from the targeted allele appears to be compromised, probably due to the selection marker unit inserted in the artificial intron of the FLIP cassette.

## cKO *via* Conditional Protein Degradation

Conditional induction of target protein degradation is another method for generating cKO cells (Figure 1E). Several techniques for conditional depletion of target proteins have been developed using mutant FKBP, Halo-tag, and auxin-inducible degron (AID) as tag-sequences, and the small chemical compounds, Shld1, HyT13, and auxin [indole-3-acetic acid (IAA)], as regulators of degradation (Banaszynski et al., 2006; Nishimura et al., 2009; Neklesa et al., 2011). Among these approaches, the AID system is the most well-validated, and uses an *Oryza sativa*-derived TIR1 protein, which forms an E3 ubiquitin ligase complex that can induce regulated and rapid degradation of proteins fused with a 7 kDa degron tag derived from *Arabidopsis thaliana* IAA17, in a manner dependent on the small chemical compound, auxin. Thus, introduction of an AID-tag into a target gene in TIR1-expressing cells enables cKO of the target gene.

A recent report described one-step generation of degron-based cKO cells using an improved AID system, which employs mutated TIR1 and an auxin-derivative, 5-Adamantyl-IAA (5-Ad-IAA), to enhance sensitivity (Nishimura et al., 2020). cKO cells are prepared by disrupting the target gene with CRISPR/Cas9 and inserting a vector encoding the mutated TIR1 and AID-tagged target gene cDNA, connected with an internal ribosome entry sequence (IRES), into the DSB site. The targeting efficiency was approximately 75% when this system was used for conditionally knocking out a single allele gene *CENP-H*, which locates on the Z chromosome in DT40 cells. This system is superior to the conditional genetics in terms of reversibility and fast kinetics. Therefore it is suitable for analysis of genes that require rapid depletion, such as cell cycle-related genes. A drawback of this approach is that expression of the target gene is controlled by the IRES sequence; thus target gene expression in the cKO cells does not reflect endogenous gene expression. This is also applicable to the genes in which regulatory elements in introns are eliminated. Another concern is that depletion of the target protein could be incomplete, due to the principles underpinning the system (Ng et al., 2019).

## The All-In-One cKO Method

**FIGURE 2:**
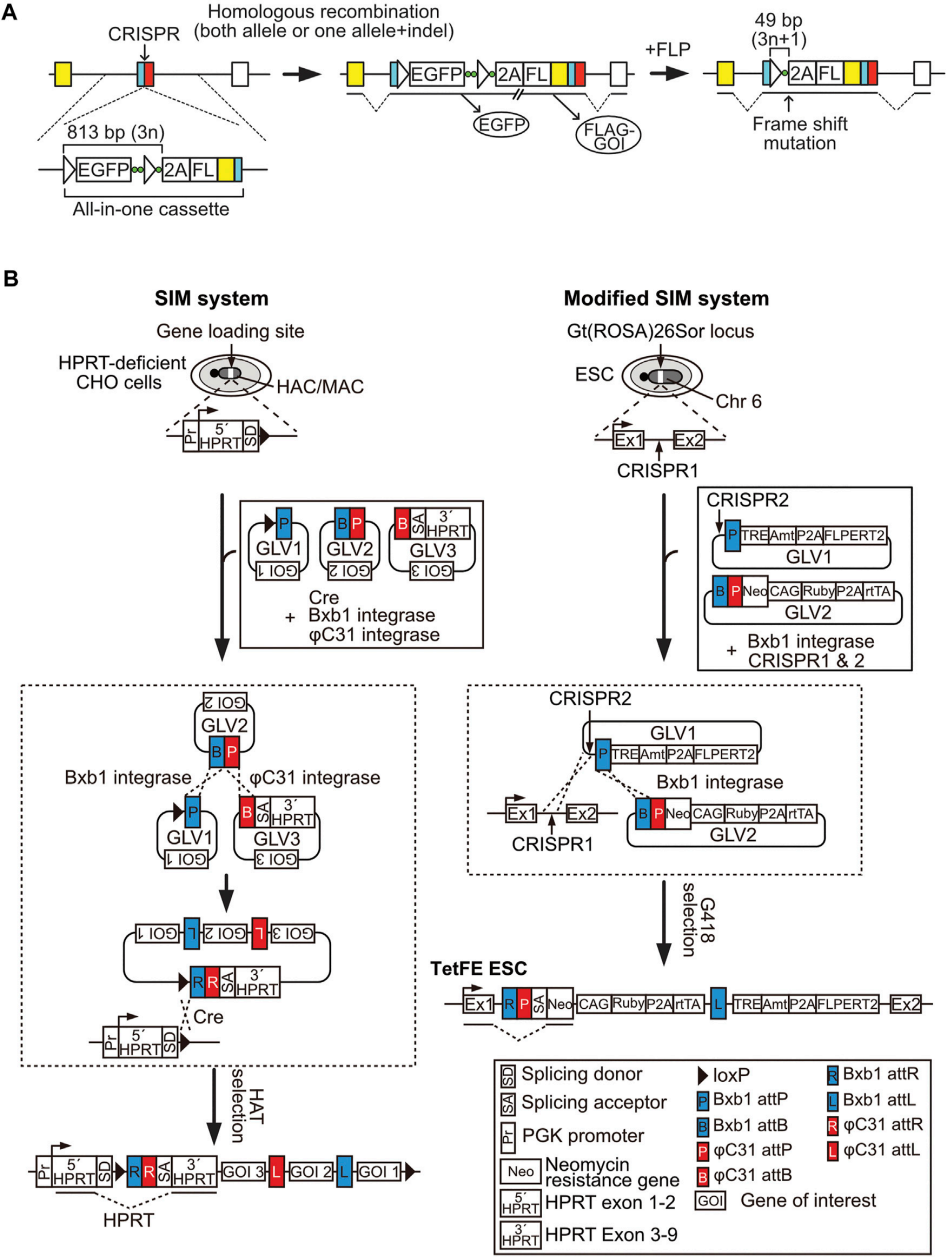
The simultaneous or sequential integration of multiple genes (SIM) system and all-in-one conditional knockout (cKO) methods. **(A)** All-in-one cKO method. Green circle, nucleotides used to induce a frame-shift mutation; empty triangle, FRT site; FL, 3 × FLAG-tag sequence. **(B)** Schematic representation of the SIM system and the modified SIM system. The SIM system and the modified SIM system enable simultaneous loading of multiple gene loading vectors (GLVs) into a human artificial chromosome/mouse artificial chromosome vector and a safe harbor locus, respectively. The procedure to generate TetFE ESCs is shown as an example for loading multiple GLVs via the modified SIM system.

The recent study reported a novel cKO method, the all-in-one cKO method, which allows one-step and highly efficient cKO and simultaneous target gene modifications, including epitope tagging and reporter gene knockout/in, *via* CRISPR/Cas9-assisted homologous recombination of the all-in-one cassette in a coding exon of the target gene (Figure 2A) (Suzuki et al., 2021). The all-in-one cassette encodes an FRT-flanked promoter-less *EGFP* gene, followed by a P2A peptide sequence, a FLAG-tag sequence, and the coding sequence of the target gene, upstream of the CRISPR/Cas9 target site. Since the EGFP cassette does not contain a promoter, sorting of EGFP-positive cells enables efficient isolation of cKO mESC clones at a frequency of up to 80%. Moreover, targeting of the all-in-one cassette in the presence of the DNA-PK inhibitor, M3814, which enhances homologous recombination, followed by EGFP sorting, resulted in almost 100% cKO efficiency, even in the recombination-non-proficient human fibrosarcoma cell line, HT1080 (Riesenberg et al., 2019). Given this high efficiency, homozygous targeted cKO clones could be easily isolated, even from HT1080 cells. Target gene expression can be monitored *via* EGFP expression in cKO cells, and protein expression can be detected using an anti-FLAG antibody; this feature greatly improves the detection sensitivity of target proteins by western blotting or immunocytochemistry, and is useful for conducting chromatin immunoprecipitation (ChIP) and crosslinking immunoprecipitation (CLIP) assays, as well as for affinity purification of binding proteins for mass spectrometric analysis.

In addition, to enable instant and strictly regulated induction of KO cells from cKO cells, a TetFE ESC line was established. TetFE ESCs have an rtTA expression cassette and a tetracycline response element-regulated FLPERT2 expression cassette in the Gt (ROSA)26Sor locus to maintain stable expression of transgenes. A simultaneous or sequential integration of multiple gene loading vectors (SIM) system and modified SIM system were employed to introduce those genes (Figure 2B). The SIM system was originally developed for efficient sequential introduction of unlimited number of gene loading vectors (GLVs) or simultaneous introduction of multiple GLVs into human/mouse artificial chromosomes (HAC/MAC) (Suzuki et al., 2014; Uno et al., 2018; Suzuki et al., 2020). Both the SIM system and the modified SIM system uses gene-loading modules called SIM cassettes, which contain recognition sequences for Bxb1 and/or φC31 recombinases/integrases, to combine a maximum of three GLVs. While the SIM system utilizes the Cre/loxP reaction for integration of the combined GLVs to the gene-loading site of HAC/MAC, the modified SIM system employs CRISPR/Cas9 for integration to a safe harbor locus *via* NHEJ-dependent knock-in (Figure 2B). Co-transfection of the GLVs and recombinases/integrases expression vectors to 3 × 10^5^ target cells was sufficient for obtaining correctly recombined cell clones (Suzuki et al., 2014). It is important to know that human and mouse genomes contain pseudo-attP sequences, which are recognized by φC31 integrase (Thyagarajan et al., 2001). Therefore, validation of GLV integration to an intended site is essential for utilizing the modified SIM system. TetFE ESCs stably express rtTA and conditionally express FLP with an ERT2 domain (FLPERT2), which enables 4-hydroxytamoxifen (4-OHT)-dependent nuclear localization in a Dox-dependent manner. Therefore, KO cells can be easily induced *via* addition of Dox and 4-OHT. This dual regulation system completely prevents spontaneous KO induction caused by leaky activity of FLPERT2 and background expression of Dox-regulated genes. This drug-inducible feature is also advantageous for large-scale preparation of KO cells.

Application of the all-in-one cKO system in conjunction with the TetFE ESCs has been demonstrated in functional analysis of the RNA helicase, DDX1, in ESCs. DEAD box RNA-helicases contain characteristic Asp-Glu-Ala-Asp (DEAD) box motifs that are involved in various RNA metabolism processes, including translation, pre-tRNA splicing, and ribosomal biogenesis (Linder and Jankowsky 2011). DDX1 is a DEAD box RNA helicase suggested to be involved in viral replication, tRNA synthesis, and miRNA processing (Fang et al., 2004; Han et al., 2014; Popow et al., 2014). Further, loss of *Ddx1* stalls mouse development at the 2 to 4 cell stage, possibly due to mis-regulation of DDX1-bound mRNA, which is crucial for 1 to 4 cell stage embryo development (Hildebrandt et al., 2019); however, the precise molecular mechanisms underlying these functions have not been fully elucidated. To clarify these molecular functions, we prepared *Ddx1* cKO TetFE ESCs and found that loss of *Ddx1* resulted in a severe growth defect. Furthermore, the number of TUNEL-positive apoptotic cells was significantly increased in *Ddx1* ^*−/−*^ ESCs. Accordingly, expression of p53, the master-regulator of cell growth and survival, was upregulated in *Ddx1* ^*−/−*^ ESCs. These results indicated that loss of *Ddx1* activated the p53-signaling pathway. Further analysis revealed that loss of DDX1 led to an rRNA processing defect, resulting in p53 activation *via* a ribosome stress pathway. Consistent with these findings, the apoptotic phenotype of *Ddx1* ^*−/−*^ ESCs was rescued by disruption of the *p53* gene. These molecular analyses illustrate the practical utility of the all-in-one cKO method, while most recently developed cKO methods have only been used for proof-of-principle experiments. The disadvantage of the all-in-one cKO method is that it is only applicable for genes whose promoters can drive detectable levels of EGFP expression. Further improvements will be essential to allow application of the method for genes with low expression levels.

## Discussion

The recent development of new strategies has greatly reduced the time and labor required for preparation of cKO cells. To encourage wider use of these cKO methods in the academic community, further improvements may be needed. While methods developed to date allow generation of cKO cells in a single step, most still require relatively large-scale screening to isolate a clone due to low cKO efficiencies of approximately 5%. Moreover, some CRISPR/Cas9-assisted methods rely on disruption of untargeted alleles (Figures 1D,E), which could restrict the utility of the cKO cells, since cKO cells obtained by this method express lower levels of the target gene than the parental cells, as the target gene is only expressed from the targeted allele. To overcome these limitations, improvements in targeting rate are required for efficient preparation of homozygous targeted cKO clones. Application of homologous recombination-enhancing drugs/genes may improve efficiency, as demonstrated in the all-in-one cKO method. Further, production of homozygous targeted cKO clones in aneuploid cell lines should further broaden the utility of these approaches. The procedure for KO induction of cKO cells is also a potential hurdle to general application of these methods. Since stable expression of recombinases is potentially toxic to cells, transient recombinase expression is appropriate for KO induction. Drug-inducible recombinase expression systems would also be convenient, and are used in several methods. The Dox-inducible, 4-OHT-regulated system applied in the all-in-one cKO method may be optimal for strict regulation of KO induction, as it avoids background induction of KO cells, due to leaky expression/activity of KO inducers.

Conditional knockout of multiple genes can be useful for analysis of signaling pathways or functions of redundant genes. Although simultaneous cKO of two genes was demonstrated using the inducible Cas9-mediated cKO system, the double KO efficiency was 40–50%, which is insufficient to allow molecular function analysis in most cases. Moreover, the system cannot induce KO of each gene separately. Production of cKO cell lines by applying multiple-recombinases and their recognition sequences *via* the efficient methods reviewed here could resolve these issues.
